# Molecular identification of blood meal sources of ticks (Acari, Ixodidae) using cytochrome b gene as a genetic marker

**DOI:** 10.3897/zookeys.478.8037

**Published:** 2015-01-28

**Authors:** Ernieenor Faraliana Che Lah, Salmah Yaakop, Mariana Ahamad, Shukor Md Nor

**Affiliations:** 1 Acarology Unit, Infectious Diseases Research Centre, Institute for Medical Research, Jalan Pahang, 50588, Kuala Lumpur, Malaysia; 2 School of Environmental and Natural Resource Sciences, Faculty of Sciences and Technology, University Kebangsaan Malaysia, 43600 Bangi, Selangor, Malaysia

**Keywords:** Ticks, Blood meal, Vector control, hosts, Cytochrome b

## Abstract

Blood meal analysis (BMA) from ticks allows for the identification of natural hosts of ticks (Acari: Ixodidae). The aim of this study is to identify the blood meal sources of field collected on-host ticks using PCR analysis. DNA of four genera of ticks was isolated and their cytochrome b (Cyt *b*) gene was amplified to identify host blood meals. A phylogenetic tree was constructed based on data of Cyt *b* sequences using Neighbor Joining (NJ) and Maximum Parsimony (MP) analysis using MEGA 5.05 for the clustering of hosts of tick species. Twenty out of 27 samples showed maximum similarity (99%) with GenBank sequences through a Basic Local Alignment Search Tool (BLAST) while 7 samples only showed a similarity range of between 91–98%. The phylogenetic trees showed that the blood meal samples were derived from small rodents (*Leopoldamys sabanus*, *Rattus tiomanicus* and *Sundamys muelleri*), shrews (*Tupaia glis*) and mammals (*Tapirus indicus* and *Prionailurus bengalensis*), supported by 82–88% bootstrap values. In this study, Cyt *b* gene as a molecular target produced reliable results and was very significant for the effective identification of ticks’ blood meal. The assay can be used as a tool for identifying unknown blood meals of field collected on-host ticks.

## Introduction

The tick is a member of the class Arachnida that belongs to the sub-class Acari. It relies heavily on other animals as hosts to complete their life cycle (Mihalca et al. 2012). The tick is the second important vector after mosquitoes and is able to transmit disease in both man and livestock (Dalgic et al. 2010, Smith et al. 2010). Ticks may transmit a range of disease agents that are of medical and veterinary importance when taking a blood meal. Ticks that live in close proximity to humans, especially those on rodents, play a significant role in the transmission of several diseases (Chul-Min et al. 2006). Besides their role as hosts, rodents also serve as reservoirs of tick-borne pathogens (Paulauskas et al. 2009, Harrison et al. 2010, Paziewska et al. 2010). The close association between rodents and human is a risk factor for the transmission of diseases such as rickettsiosis, babesiosis and Lyme borreliosis (Killilea et al. 2008, Kia et al. 2009, Mihalca et al. 2012).

Research on the identification of a natural host for ticks is valuable and is the main goal of a blood meal analysis (BMA). The analysis is a tool used to identify the hosts of blood feeding arthropods (Gariepy et al. 2012). Research into the composition of blood meals of arthropod vectors has been shown to provide evidence of link vector species with their specific hosts that can be used to determine possible disease reservoirs (Scott et al. 2012, Onder et al. 2013). Knowledge of vector host identification of ticks is critical in understanding the transmission cycle of vector-borne diseases (Christine 2011) and thus can provide important information to public health stakeholders for the development of more effective control strategies. Initially, attempts to identify blood meals of arthropods were made using serological techniques such as precipitin and latex agglutination tests (Walker and Davies 1971, Nevil and Anderson 1972). An improvement on the sensitivity of blood meal origin analysis was later studied using enzyme-linked immunosorbent assays (ELISA) (Elias et al. 2010, Maria Stella et al. 2012). While those techniques continue to provide valuable and insightful data, the identification of many blood meal sources is limited to just the order or family level and species could only be identified when antibodies were available. The advert of the polymerase chain reaction (PCR) and the availability of DNA sequence data of various vertebrates quickly led to a shift towards DNA-based identification (Mukabana et al. 2002, Humair et al. 2007, Kent 2009, Garros et al. 2011).

The progressive development of molecular analysis has resulted in the use of markers that were originally designed to study phylogenetic relationships among vertebrates being applied to determine the blood meal origin of haematophagous arthropods (Boakye et al. 1999, Ninio et al. 2011). Tobolewski et al. (1992) reported that DNA techniques are much faster and more reliable to determine host species compared to other techniques that depend on antibodies. Humair et al. (2007) used 12S rDNA as a genetic marker for identification of blood meal sources in *Ixodes ricinus* ticks while Pichon et al. (2003) used a set of universal primers to amplify part of the vertebrate 18S rRNA gene followed by reverse line blot hybridization for host identification of *Ixodes ricinus*. Understanding the origin of blood meals will give some information on the species involved in disease transmission cycles, as well as the disease risk posed to human.

Analysis of blood meals of local ticks to determine their natural hosts is still poorly understood. There is a critical necessity to document information of potential hosts for local ticks as part of the nation’s preparedness for emerging and re-emerging infections. Thus, the aim of this study was to identify the blood meal sources of ticks in Peninsular Malaysia based on the cytochrome b (Cyt *b*) gene. The obtained Cyt *b* genes were then used to construct a phylogenetic tree for the clustering of hosts of tick species.

## Materials and methods

### Study area and ticks sampling

**Figure 1.**
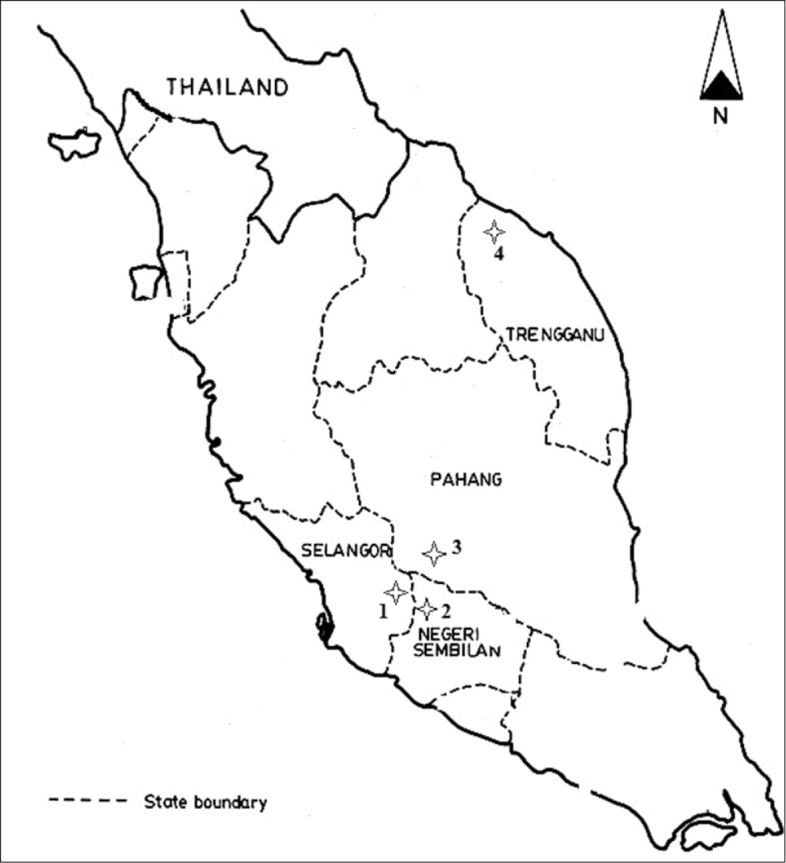
Map of the study sites in Peninsular Malaysia: **1** Hulu Langat, Selangor **2** Labu, Negeri Sembilan **3** Janda Baik, Pahang **4** Gunung Tebu, Terengganu.

On-host ticks were collected from four different states of Peninsular Malaysia (Figure 1). The study sites were in Janda Baik, Pahang; Labu, Negeri Sembilan; Hulu Langat, Selangor; and Gunung Tebu, Terengganu. The collections were conducted from March to July 2012. The localities were chosen based on the records from available previous data of high numbers of tick infestation on small animals. The ecology for all localities was similar; consisting of mainly pristine tropical lowland rainforest, secondary growths, scrubs and riverine vegetation. One hundred wire traps were used to capture wild rodents and tree-shrews in each study site. Traps were placed on the ground and on tree branches along existing trails at approximately five meter intervals. Traps were baited with bananas, oil palm fruits, tapioca or potatoes and checked once daily for 5 consecutive days of trapping. Caught animals were placed in cloth bags and brought back to the Institute for Medical Research (IMR). The animals were anesthetized with chloroform before screening and the ticks were collected in the laboratory. The epidemiology data such as locality and ecology were recorded.

### Tick Morphological identification

The species of animals were identified by their morphological traits following Medway (1983), Francis (2008) and Payne et al. (2005). A total of 27 on-host ticks were collected either using sterile soft forceps or sharpened wooden applicator sticks. The ticks were then kept individually in vials containing 70% ethanol prior to identification using specific illustrated morphological taxonomic keys (Kohl 1957, Walker et al. 2003).

### DNA extraction of blood meals in ticks

Prior to DNA extraction, each engorged tick was individually washed 3 times with sterile distilled water. Extraction of DNA using QIAamp Mini Kit (Qiagen, Germany) was performed according to the manufacturer’s protocols. DNA of ticks was extracted by adding 80 µl of PBS buffer and 100 µl of ATL buffer into the sample. The ticks were then macerated using sterile tips for 5 minutes before adding of 20 µl of proteinase K. The samples were incubated at 56 °C (6 hours) for complete lyses. The following steps were the same as those in the manufacture’s protocols. The DNA was then used for subsequent PCR.

### PCR amplification of the mtDNA Cyt *b* gene

A portion of mitochondrial DNA, Cyt *b* gene was amplified by Polymerase Chain Reaction (PCR) with a set of vertebrate-universal primers and reaction conditions as described by Kent and Norris (2005). The primers used were UNFOR403 and UNREV1025 (Table 1) that preferentially amplified a 623-bp region of the Cyt *b* gene from the mitochondrial DNA of vertebrates. The PCR reactions were conducted in 50 µl reaction tubes with the following reagents: 25 µl Taq PCR master 2X, 2.5 µl of 0.5 µM of each primer, 10 µl of nuclease free water and 10 µl of DNA template. The amplification program consists of a total of 35 cycles, denaturing at 94 °C for 3 min, annealing at 52 °C for 1 min, and extension at 72 °C for 1 min, with an initial denaturation at 94 °C for 1 min. PCR was carried out using an Eppendorf Master Cycler Personal machine (Eppendorf, Germany). For each PCR reaction, a negative control containing the DNA of unfed ticks and a positive control containing vertebrate DNA was included. The amplicons were visualized in 1.5% agarose gels stained with ethidium bromide and viewed under an ultraviolet trans-illuminator (wavelength 254 nm).

**Table 1.** List of primers used for PCR amplification

Gene	Primer name	Sequences (5’–3’)
Cyt *b*	UNFOR403	5'-TGA GGA CAA ATA TCA TTC TGA GG-3'
	UNREV1025	5'-GGT TGT CCT CCA ATT CAT GTT A-3'

### Sequencing analysis and Alignment

The PCR product was excised with a sterile gel cutter and purified using 5 Prime PCR Agarose Gel Extract Mini Kit (Hamburg, Germany) according to the manufacturer’s protocols. The purified product was then sent to the sequencing service company, Medigene Sdn. Bhd. in Petaling Jaya, Selangor. The sequencing was bi-directional for all specimens and the primer combination for this step was the same as that used in the PCR amplification. Sequencing results were exported as FASTA sequence files. The Cyt *b* gene sequences of samples were aligned using ClustalW multiple alignment of BioEdit (Thompson et al. 1994, Hall 2005).

### BLAST analysis

The obtained sequences were then compared with available sequences in the GenBank database using the Basic Local Alignment Search Tool search (NCBI website, http://www.ncbi.nlm.nih.gov/BLAST/) for the identification of the host species. This approach was reported to be simple and robust for rapid comparison of query sequences to database sequences leading to species identification (Altschul et al. 1990, Mitler et al. 2010). The approach enabled the similarity of sequences to be measured depending on several criterias such as expected value, maximum identical, query coverage and maximum score.

### Clustering analysis

The clustering analysis for all sequences of hosts was carried out by performing phylogenetic analysis using MEGA software (version 5.05). For distance analysis, a neighbor-joining (NJ) tree was generated from a Kimura two-parameter distance matrix. Maximum-parsimony (MP) analysis was performed with Tree-Bisection-Reconnection (TBR) heuristic algorithm to reconstruct a character based phylogenetic tree. Internal branches of both trees were statistically supported by bootstrapping with 1,000 replications. In this study, *Apodemus sylvaticus* (GenBank Accession no. AJ298599.1) was selected as an outgroup for Cyt *b* gene.

## Results

A total of 27 engorged ticks collected from four different localities (Table 2) were amplified from Cyt *b* using PCR analysis. Six species of hosts comprising three rodents (*Leopoldamys sabanus*, *Sundamys muelleri* and *Rattus tiomanicus*); a shrew, *Tupaia glis* and two mammals identified as *Tapirus indicus* and *Prionailurus bengalensis*, were identified. Mixed stages of on-host ticks were collected from the animals and identified into four genera: *Ixodes*, *Amblyomma*, *Haemaphysalis* and *Dermacentor*.

**Table 2.** Details of ticks samples used in analysis.

**Code samples**	**Tick species**	**Locality**	**Ecology**
K1a	*Amblyomma testudinarium*	Krau Wildlife Reserved, Pahang	Pristine tropical rainforest
K2a	*Dermacentor* sp.	Krau Wildlife Reserved, Pahang	Pristine tropical rainforest
K3c	*Dermacentor* sp.	Krau Wildlife Reserved, Pahang	Pristine tropical rainforest
K4b	*Amblyomma* sp.	Krau Wildlife Reserved, Pahang	Pristine tropical rainforest
K5b	*Amblyomma* sp.	Krau Wildlife Reserved, Pahang	Pristine tropical rainforest
SBN 01	*Ixodes granulatus*	Labu, Negeri Sembilan	Scrubs
SBN12_1	*Dermacentor* sp.	Labu, Negeri Sembilan	Scrubs
SBN23_1	*Ixodes granulatus*	Labu, Negeri Sembilan	Scrubs
SBN 14	*Ixodes* sp.	Labu, Negeri Sembilan	Scrubs
JBB01_1	*Dermacentor* sp.	Janda Baik, Pahang	Riverine vegetation
JBB03_1	*Haemaphysalis* sp.	Janda Baik, Pahang	Riverine vegetation
JBB03_2	*Haemaphysalis* sp.	Janda Baik, Pahang	Riverine vegetation
JBB03_3	*Haemaphysalis* sp.	Janda Baik, Pahang	Riverine vegetation
JBB03_4	*Haemaphysalis* sp.	Janda Baik, Pahang	Riverine vegetation
HL01	*Ixodes granulatus*	Hulu Langat, Selangor	Pristine tropical rainforest
HL02	*Ixodes granulatus*	Hulu Langat, Selangor	Pristine tropical rainforest
HL03_2	*Ixodes granulatus*	Hulu Langat, Selangor	Pristine tropical rainforest
HL03	*Ixodes granulatus*	Hulu Langat, Selangor	Pristine tropical rainforest
HL04_15	*Ixodes granulatus*	Hulu Langat, Selangor	Pristine tropical rainforest
HL02_4	*Ixodes granulatus*	Hulu Langat, Selangor	Pristine tropical rainforest
HL02_5	*Ixodes granulatus*	Hulu Langat, Selangor	Pristine tropical rainforest
HL02_3	*Ixodes granulatus*	Hulu Langat, Selangor	Pristine tropical rainforest
HL07	*Ixodes granulatus*	Hulu Langat, Selangor	Pristine tropical rainforest
HL02_1	*Ixodes granulatus*	Hulu Langat, Selangor	Pristine tropical rainforest
GT23_2	*Ixodes granulatus*	Gunung Tebu, Terengganu	Secondary growth
GT23_3	*Ixodes granulatus*	Gunung Tebu, Terengganu	Secondary growth
GT23_5	*Ixodes granulatus*	Gunung Tebu, Terengganu	Secondary growth

### PCR products and BLAST analysis

**Figure 2.**
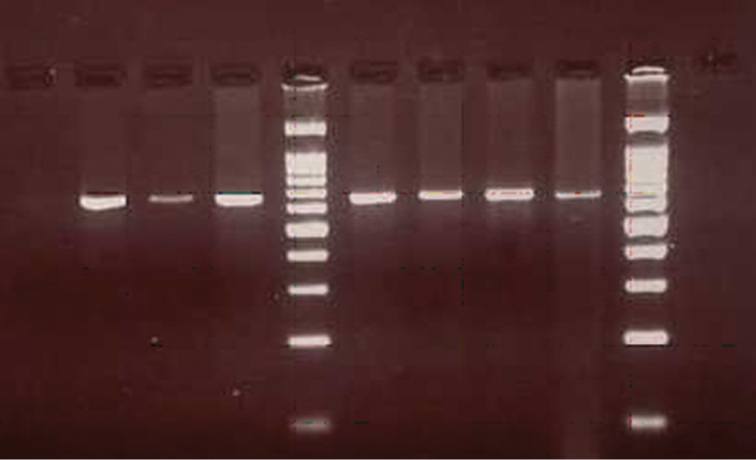
Amplification of Cyt *b* gene produced 623 bp of PCR products from ticks species. *Lane 1*: unfed ticks (negative control); *Lane 2*: vertebrate DNA (positive control); *Lanes 3–8*: DNA of field-collected ticks (*Ixodes* sp., *Dermacentor* sp., *Amblyomma* sp., *Amblyomma testudinarium*, *Haemaphysalis* sp., *Haemaphysalis* sp.,) and *Lanes* M1, M2: 100 bp DNA ladder (Bioron, Germany).

Vertebrate DNA was successfully amplified from 27 engorged ticks. The amplification of a single fragment encoding a 623 bp sequence of the Cyt *b* gene yielded the expected amplification products (Figure 2). Negative control (unfed ticks) yielded no PCR product implying that only host DNA patterns were detected in the amplifying specimens. All blood meals were identified down to species level. In general, the percent similarity between queried (unknown) sequences and the closest match in GenBank was between 91–100% (Table 3). Twenty out of 27 sequences (74.1%) showed maximum homology (99%) while 2 sequences (HL04_15 and SBN23_1) displayed 98% similarity with the online database. One sequence (HL03) showed maximum homology to *Leopoldamys sabanus* with a 97% similarity while four sequences from Janda Baik matched to *Sundamys muelleri* with a 91%. Some of the differences were probably due to intraspecific variation and in some cases, it could be due to poor sequence quality, particularly when unassigned nucleotides (n’s) were present in the sequence.

**Table 3.** Blasting results against available sequences in GenBank.

**Code samples**	**Tick species**	**Host species (morphological)**	% **similarity with GenBank (species)**
K1a	*Amblyomma testudinarium*	*Tapirus indicus*	99 (*Tapirus indicus*)
K2a	*Dermacentor* sp.	*Tapirus indicus*	99 (*Tapirus indicus*)
K3c	*Dermacentor* sp.	*Tapirus indicus*	99 (*Tapirus indicus*)
K4b	*Amblyomma* sp.	*Tapirus indicus*	99 (*Tapirus indicus*)
K5b	*Amblyomma* sp.	*Tapirus indicus*	99 (*Tapirus indicus*)
SBN 01	*Ixodes granulatus*	*Rattus tiomanicus*	99 (*Rattus tiomanicus*)
SBN12_1	*Dermacentor* sp	*Rattus tiomanicus*	99 (*Rattus tiomanicus*)
SBN23_1	*Ixodes granulatus*	*Rattus tiomanicus*	98 (*Rattus tiomanicus*)
SBN 14	*Ixodes* sp.	*Rattus tiomanicus*	99 (*Prionailurus bengalensis*)
JBB01_1	*Dermacentor* sp.	*Tupaia glis*	99 (*Tupaia glis*)
JBB03_1	*Haemaphysalis* sp.	*Sundamys muelleri*	91 (*Sundamys muelleri*)
JBB03_2	*Haemaphysalis* sp.	*Sundamys muelleri*	91 (*Sundamys muelleri*)
JBB03_3	*Haemaphysalis* sp.	*Sundamys muelleri*	91 (*Sundamys muelleri*)
JBB03_4	*Haemaphysalis* sp.	*Sundamys muelleri*	91 (*Sundamys muelleri*)
HL01	*Ixodes granulatus*	*Leopoldamys sabanus*	99 (*Leopoldamys sabanus*)
HL02	*Ixodes granulatus*	*Leopoldamys sabanus*	99 (*Leopoldamys sabanus*)
HL03_2	*Ixodes granulatus*	*Leopoldamys sabanus*	99 (*Leopoldamys sabanus*)
HL03	*Ixodes granulatus*	*Leopoldamys sabanus*	97 (*Leopoldamys sabanus*)
HL04_15	*Ixodes granulatus*	*Sundamys muelleri*	98 (*Sundamys muelleri*)
HL02_4	*Ixodes granulatus*	*Leopoldamys sabanus*	99 (*Leopoldamys sabanus*)
HL02_5	*Ixodes granulatus*	*Leopoldamys sabanus*	99 (*Leopoldamys sabanus*)
HL02_3	*Ixodes granulatus*	*Leopoldamys sabanus*	99 (*Leopoldamys sabanus*)
HL07	*Ixodes granulatus*	*Leopoldamys sabanus*	99 (*Leopoldamys sabanus*)
HL02_1	*Ixodes granulatus*	*Leopoldamys sabanus*	99 (*Leopoldamys sabanus*)
GT23_2	*Ixodes granulatus*	*Leopoldamys sabanus*	99 (*Leopoldamys sabanus*)
GT23_3	*Ixodes granulatus*	*Leopoldamys sabanus*	99 (*Leopoldamys sabanus*)
GT23_5	*Ixodes granulatus*	*Leopoldamys sabanus*	99 (*Leopoldamys sabanus*)

### Clustering inferens

From the 27 aligned DNA sequences, a total of 575-bp portion of the Cyt *b* gene was used for analysis. Out of 575 characters from Cyt *b* fragments, 252 variable sites were detected, among which 203 (35.3%) variable characters were parsimony-informative while 49 (8.5%) characters were parsimony-uninformative. Additionally, the conserved sites were constituted by 323 (56.1%) characters showing that Cyt *b* gene is a very conserved gene in the mtDNA.

**Figure 3.**
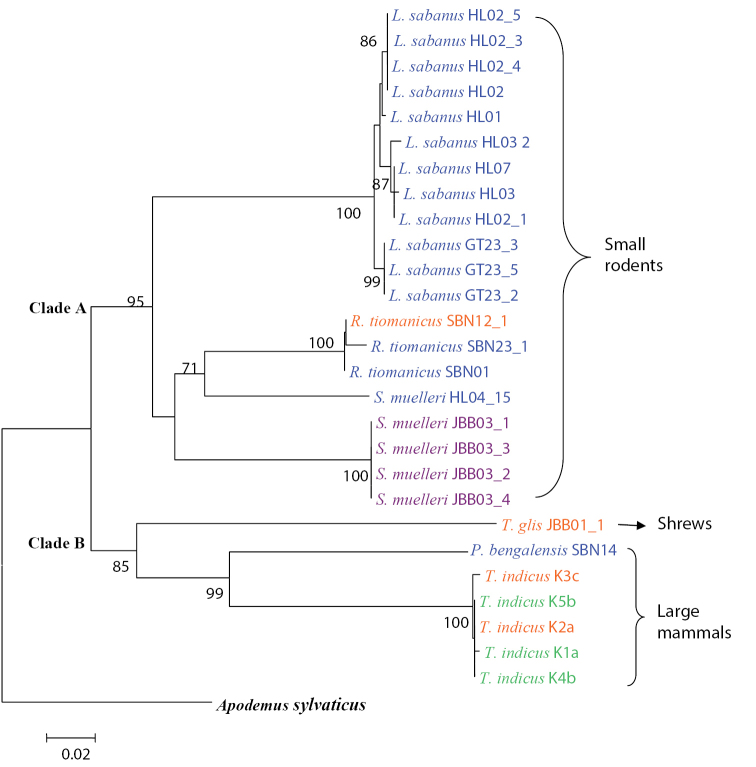
Neighbor-joining tree constructed from 28 sequences (including one outgroup sequence) of the Cyt *b* gene. The numbers at the branches stand for bootstrap values 70% and above of 1000 replications. Genera of ticks represented by blue for *Ixodes* sp., orange for *Dermacentor* sp., violet for *Haemaphysalis* sp. and green for *Amblyomma* sp.

The mitochondrial Cyt *b* gene sequences of host species were grouped into 2 major clades by NJ and MP analysis; one group of small rodents and another consisting of shrews and large mammals. The tree topology showed that all 27 host sequences examined fell into two distinct genetic lineages: Clade A (consists of *Leopoldamys sabanus*, *Rattus tiomanicus* and *Sundamys muelleri*) and Clade B (consists of *Tupaia glis*, *Prionailurus bengalensis* and *Tapirus indicus*). NJ tree topology revealed a distinction with 95% bootstrap value for Clade A but a lower bootstrap value of 85% for Clade B (Figure 3). Significant grouping of *Leopoldamys sabanus*, *Rattus tiomanicus* and *Sundamys muelleri* in each independent monophyletic subclade was obtained with a 100% bootstrap value. The NJ tree also showed the infestation of *Ixodes* ticks on 3 species of different hosts that is *Leopoldamys sabanus*, *Rattus tiomanicus* and *Prionailurus bengalensis* while the *Haemaphysalis* ticks infested only on a single host, *Sundamys muelleri*.

**Figure 4.**
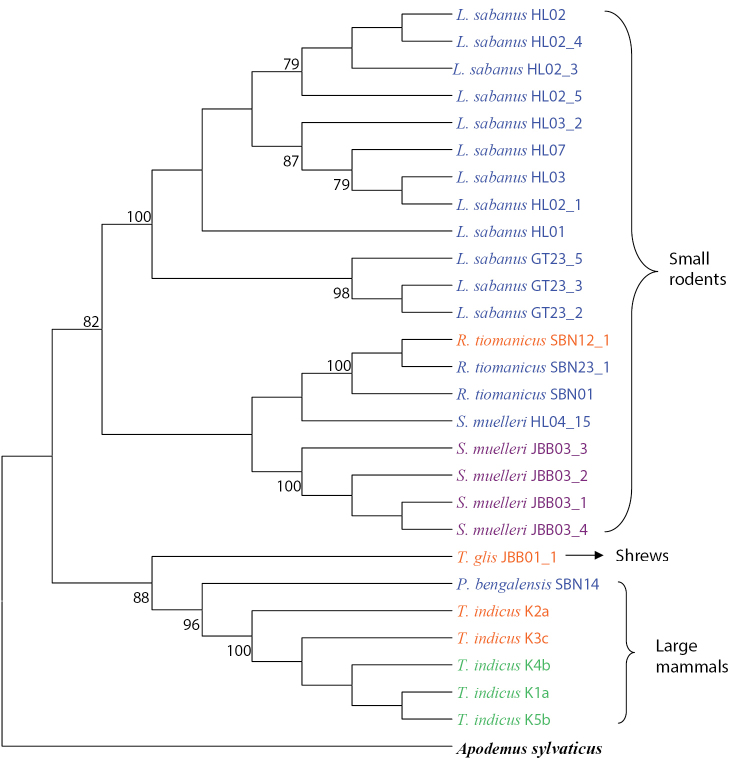
Maximum parsimony tree constructed from 28 sequences (including one outgroup sequence) of the Cyt *b* gene. The numbers at the branches stand for bootstrap values 70% and above of 1000 replications. Genera of ticks represented by blue for *Ixodes* sp., orange for *Dermacentor* sp., violet for *Haemaphysalis* sp. and green for *Amblyomma* sp.

Seven parsimonious trees were produced by the MP analysis using equally weighted TBR. The best tree had 497 steps (Figure 4) with a consistency index of 0.67114, a homoplasy index of 0.70422 and a retention index of 0.88804. The MP tree is concordant with the NJ tree as it shares a relatively similar tree topology of phylogenetic relationships. Two distinct monophyletic clades are clearly shown on the phylogenetic tree of potential hosts of ticks. These small rodents and large mammals were fully supported by high bootstrap values of 82% and 88%, respectively.

## Discussion

The study produced the first analysis of host identification for local ticks that identifies animals to the species level by detecting their Cyt *b* gene in engorged on-host ticks. The usefulness of blood meal analysis in determining the identity of the host species by PCR has been demonstrated by a recent study (Ernieenor et al. 2012). The identification of the vertebrate hosts using molecular detection of the Cyt *b* gene displayed an exact match and this adds more confidence to the use of this analysis. In the present case, all the samples were conclusively identified to species level. The results of this study also indicated that local ticks are not host specific because they have a wide host range from small rodents to mammals.

In this study, the Cyt *b* gene was successfully proven as a discriminatory molecular marker for the identification of host DNA in ticks. Cyt *b* was selected as the target gene because of its track record in blood meal identification assays and its utilization in developing mammalian phylogeny (Kirstein and Gray 1996, Pierce et al. 2009). Moreover, the Cyt *b* gene has been widely used to make reliable blood meal identification of arthropods inclusive ticks due to its high copy numbers as mitochondrial genes and sufficient genetic variations at the primary sequence level among vertebrate taxa (Ngo and Kramer 2003, Maleki-Ravasan et al. 2009).

The most significant aspect of the method is its sensitivity to detect minuscule amounts of host DNA. In this study, host DNA could be detected in the 27 engorged ticks as determined by electrophoresis on agarose gel. The successful identification presumably because ticks sample was in the freshly engorged state, sufficient intact host DNA was present in the midgut. Adult stage of the ticks was used in this analysis may also eventually give positive identification of host. Cadenas et al. (2007) had similar complications, reporting that the ability to detect host DNA in adult ticks was significantly higher than in nymphs, probably due to the higher quantity of blood ingested by ticks during their previous bloodmeals as nymphs and larvae.

This study shows that *Ixodes granulatus* may probably pose greater problems than most other ticks because of its capability to infest various hosts such as rodents and larger animals. This finding is in accordance with studies that reported *Ixodes granulatus* as the most notable tick infesting rodents (Mariana et al. 2008, Nadchatram 2008, Paulauskas et al. 2011, Mihalca et al. 2012, Mihalca and Sandor 2013). The species is a common acarine ectoparasite of rodents in Malaysia with wide distribution extends from Southeast Asia to eastern India and China (Nadchatram 2008). *Ixodes* are the most common genus of ticks that feed on humans (Briciu et al. 2011) may also infest rodents that can serve as reservoir hosts for human diseases (Marchette 1966, Schorn et al. 2011). Several genera of ticks found feeding on one individual of host is a common feeding behavior in wild rodents (Mariana et al. 2005). The sharing of host by different genera or species of ticks is important mainly for the bridging of microbial pathogens through reservoir hosts (Bursali et al. 2010). The ecological importance of reservoir hosts is greater if the animals are also common hosts for competent ticks (Mihalca et al. 2012).

An interesting finding was observed in this study where *Ixodes* ticks (SBN 14) collected from *Rattus tiomanicus* gave a high similarity (99%) of blood meal to *Prionailurus bengalensis*. It was probably due to incomplete or an interruption of feeding that occurred before the tick attached itself to another host (Allan et al. 2010). Another possible reason is the short duration of feeding on the first host before the partially fed ticks dropped off-host and attached to a new host (*Prionailurus bengalensis*) for a blood meal. A similar finding known as co-feeding transmission was also reported by Gern and Rais (1996) and Shih and Spielman (1993).

The finding of *Tapirus indicus* as one of the hosts for *Amblyomma* ticks (K1a, K4b and K5b) was rare because the ticks were generally host specific on wild reptiles and amphibians (Pandit et al. 2011). That appears to indicate that the population of reptiles and amphibians surrounding the locality are scarcely found and the ticks have no choice to feed on any other animals available in the area. Rodents such as *Leopoldamys sabanus*, *Rattus diardii* and *Niviventer rapit* were also reported as alternative hosts for *Amblyomma* ticks (Nadchatram et al. 1966, Paramasvaran et al. 2009).

Partial Cyt *b* mtDNA gene sequences used in this study seems to be effective in identifying the phylogenetic relationships between ticks and their hosts. This is because both NJ and MP tree topology showed very clear distinction between groups of small rodents, shrews and large mammals with a highly supported monophyletic clade. In small rodents, the node is solved in the NJ and MP tree as two monophyletic clades, representing the species of *Leopoldamys sabanus*, *Rattus tiomanicus* and *Sundamys muelleri*. The tree topologies also show that *Tupaia glis* formed their own distinct monophyletic clade separated from other larger mammals which consists of *Prionailurus bengalensis* and *Tapirus indicus*. The information obtained has further corroborated the morphological identification and classification of *Tupaia glis* which comes under the group of shrews. Such knowledge gained through host preference studies is essential to understanding the relationship of host and vector and their roles in the enzootic transmission cycle (Ngo and Kramer 2003). Therefore, analyses of ticks bloodmeals will create a better understanding of epidemiologically important vectors-their hosts that can be lead to the design of more effective control strategies (Kirstein and Gray 1996).

Of particular interest was the high numbers of ticks infesting small rodents compared to larger mammals. This finding is in agreeable with a previous study which reported that the abundance of small rodents compensate even if the intensity of tick parasitism on them is smaller than the larger hosts (Randolph 2004). The capability of rodents as host for ticks has been extensively investigated until to date mainly because they can be easily captured in large numbers and are easier to handle or maintain in the laboratory (Gern and Humair 2002). This is in line with our catch which was represented mainly by rodents as they were abundantly found in all localities.

For further studies, it is recommended that ticks are collected from sites frequently visited by animals including the wallows, wildlife main trails and river banks to include a more diverse group of animals in order to generate better results (Pichon et al. 2005, Pierce et al 2009). A bigger sample size with heterogeneous host species collection needs to be examined in parallel with different molecular markers to enable researchers to draw reliable conclusions and provide alternative views on the relationship between ticks and their natural hosts in Malaysia.

## Conclusion

The PCR direct sequencing system using vertebrate Cyt *b* gene is a potential screening tool for the identification of ticks’ blood meal. The Cyt *b* gene was selected for this study based on their higher nucleotide variations for the effective identification of hosts. Blood meal identification of field collected ticks by molecular methods offer a direct and efficient approach for understanding the contributions of both competent and incompetent hosts for the transmission dynamics of tick-borne diseases. Furthermore, this valuable information can confirm a strong association between hard ticks and hosts (especially rodents) and this will assist public health officials with efforts to outline an effective tick-borne diseases control program.
